# To see, hear, and live: 25 years of the vitamin A programme in Nepal

**DOI:** 10.1111/mcn.12954

**Published:** 2020-02-27

**Authors:** Andrew L. Thorne‐Lyman, Kedar Parajuli, Naveen Paudyal, Stanley Chitekwe, Ram Shrestha, Dibya Laxmi Manandhar, Keith P. West

**Affiliations:** ^1^ Center for Human Nutrition, Department of International Health Johns Hopkins Bloomberg School of Public Health Baltimore Maryland; ^2^ Nutrition Section, Family Welfare Division Ministry of Health and Population Nepal Kathmandu Nepal; ^3^ UNICEF Nepal Kathmandu Nepal; ^4^ Nepali Technical Assistance Group Kathmandu Nepal

**Keywords:** childhood infections, child nutrition, child public health, International Child Health Nutrition, programme evaluation, vitamin A

## Abstract

Nepal has a rich history of vitamin A research and a national, biannual preschool vitamin A supplementation (VAS) programme that has sustained high coverage for 25 years despite many challenges, including conflict. Key elements of programme success have included (a) evidence of a 26–30% reduction in child mortality from two, in‐country randomized trials; (b) strong political and donor support; (c) positioning local female community health volunteers as key operatives; (d) nationwide community mobilization and demand creation for the programme; and (e) gradual expansion of the programme over a period of several years, conducting and integrating delivery research, and monitoring to allow new approaches to be tested and adapted to available resources. The VAS network has served as a platform for delivering other services, including anthelmintic treatment and screening for acute malnutrition. We estimate that VAS has saved over 45,000 young lives over the past 15 years of attained national coverage. Consumption of vitamin A‐ and carotenoid‐rich foods by children and women nationally remains low, indicating that supplementation is still needed. Current challenges and opportunities to improving vitamin A status include lower VAS coverage among younger children (infants 6–11 months of age), finding ways to increase availability and access to dietary vitamin A sources, and ensuring local programme investments given the recent decentralization of the government.

**Key messages**

- Vitamin A supplementation reduces childhood mortality, blindness, and hearing loss in Nepal.
- Nepal's vitamin A supplementation programme has maintained coverage rates >80% for 25 years, despite geographical challenges and civil conflict.
- Central to this success was placing female community health volunteers and mothers' groups at the centre of the programme, along with stakeholder cooperation and community demand creation
- Since reaching full national coverage in 2002, vitamin A supplementation is estimated to have saved the lives of over 45,000 children in Nepal.
- Consumption of dietary sources of vitamin A remains low despite surveys showing high knowledge about the dietary sources of vitamin A. Supplementation will continue to be needed until dietary intake improves.

## 1 INTRODUCTION

The history of vitamin A deficiency prevention in Nepal, through to the present day, is one of gathering evidence of the extent and severity of vitamin A deficiency, testing the impact of interventions on child mortality, forging policies with strong political support, and building, evaluating, and adapting collaborative programmes to be sustainable and effective. Vitamin A deficiency is endemic in South Asia, including Nepal, and is a fully preventable cause of xerophthalmia and nutritional blindness; mortality from severe infection, including measles and diarrhoea; anaemia; and hearing loss from severe ear infection (Ross, 1996; Schmitz et al., 2012; Sommer & West, 1996). Underlying these “vitamin A deficiency disorders” are numerous pathways and clinical observations that reveal roles for vitamin A in maintaining epithelial, immune, and haematopoietic function and provide a clear biological basis for both these consequences and their prevention (Palmer, Darnton‐Hill, & West, 2017). In the early 1970s, national vitamin A programmes were launched in India and Bangladesh to prevent xerophthalmia and consequent blindness that involved the delivery of high‐dose vitamin A capsules (200,000 international units [IUs] and half‐dose for infants) twice a year to preschool‐aged children (World Health Organization, 1976). Subsequently, the role of periodic vitamin A supplementation in preventing preschool child mortality became evident in the Aceh study, a community randomized controlled trial in 450 villages in northern Sumatra, Indonesia, from 1982–1984, which observed a 34% reduction in all‐cause mortality attributable to the vitamin A programme (Sommer et al., 1986).

In the latter half of the 1980s, Nepal became the setting for replicative vitamin A supplementation trials, prompted by reports of high child mortality rates and endemic xeropthalmia in two national surveys in 1981 (Brilliant et al., 1985; Upadhyay, Gurung, Pillai, & Nepal, 1985), which suggested that vitamin A deficiency was a serious public health problem in the country. As the country hosted the 13th meeting of the International Vitamin A Consultative Group in November 1989, two community trials were launched to test whether periodic high‐dose vitamin A supplementation could reduce child mortality in Nepal. In the *Terai* district of Sarlahi, under the auspices of Nepal Netra Jyoti Sangh (National Society for the Prevention of Blindness), a double‐masked, placebo‐controlled trial was carried out through the Johns Hopkins Nepal Nutrition Intervention Project, Sarlahi, enrolling, dosing, and following 28,630 children 6‐72 months of age. Published in 1991, the trial revealed a 30% reduction in mortality among children who received the vitamin A versus placebo supplement (relative risk (RR) 0.70, 95% confidence interval [CI] [0.56, 0.88]; West et al., 1991). Shortly after, a second trial in the Far Western Hill district of Jumla reported a similar 26% reduction in mortality among children living in randomized programme subdistricts who received a large dose of vitamin A versus children in control subdistricts (RR 0.74, 95% CI [0.55, 0.99]; Daulaire et al., 1992). Additional trials of varied design were carried out in India, Sudan, and Ghana. When combined in a meta‐analysis, the findings from a total of eight large trials carried out in Southern Asia and sub‐Saharan Africa indicated that vitamin A provision, via supplements or food fortification, could reduce preschool child mortality by, on average, 23% (Beaton et al., 1993).

Informed and guided by sound, population evidence of an impact on child survival (and blindness prevention), government policymakers, United Nations (UN), and bilateral agencies, NGOs, scientists, and donors convened in 1992 to develop plans for a national vitamin A programme. Within a year, a semiannual vitamin A supplementation campaign strategy was launched, drawing on the experiences and capabilities of an existing cadre of trained female community health volunteers (FCHVs) in every ward (a subdistrict unit) of the country. Starting in eight of Nepal's 75 districts, the programme was regularly evaluated and adapted to meet resources and needs of communities in the *Terai*, hills and mountains, under the guidance of the Nepali Technical Assistance Group (NTAG). By 2002, the programme was fully scaled up and routinely reached ~85% of all children 6–59 months of age (Ministry of Health [MOH] Nepal & ORC Macro, 2002; Ministry of Health and Population [MoHP] Nepal & Macro International, 2007; MOH Nepal & ICF, 2017). Thus launched, the programme has continued to function, be evaluated, and has been found to save numerous lives of children over the intervening years (Thapa, Choe, & Retherford, 2005).

This manuscript, developed as part of a series of papers reflecting on 25 years of Nepal's progress in nutrition, has two main objectives. The first is to describe the critical role that Nepal has played globally in scientific discovery related to vitamin A and how the country overcame clear challenges to create a programme that would be internationally lauded for its sustained high coverage over time. The second is to address the contemporary opportunities and challenges facing the country as it weighs future options to combat vitamin A deficiency.

## 2 METHODS

A symposium was held from November 27–29, 2018 to reflect on 25 years of progress implementing nutrition programmes in Nepal, including vitamin A, with a keynote speech by Professor Keith P. West Jr. and a panel discussion (Feed the Future Nutrition Innovation Lab & UNICEF, 2019). Reflections from the symposium's session on vitamin A were augmented by discussions with 30 people directly involved in policy development, programme implementation, and research, over the history of the programme, including FCHVs. Literature searches were conducted using PubMed and Google Scholar with the keywords “vitamin A” and “Nepal” to identify additional research studies and grey literature describing the programme. We also reviewed the findings of 28 minisurvey reports published from 1993 to 2010 that provided detailed analyses describing the challenges and successes encountered as the programme expanded to cover the entire country (NTAG, 1999).

We generated estimates of (a) the number of lives saved of children aged 6–59 months from 2002–2017 due to the preventive vitamin A supplementation programme from 2002, the year that the programme achieved national coverage, to 2017; and (b) mortality rates among children aged 6–59 months had the vitamin A supplementation programme not been put into place (Figure S1). To generate these estimates, we relied on modelled mortality data over time from the United Nations Interagency Group for Mortality Estimation (2018) estimates of vitamin A supplementation coverage from Demographic and Health Surveys and national micronutrient surveys and an approximate all‐cause mortality efficacy estimate of 30% on the basis of the findings from the efficacy trials in Nepal (Daulaire et al., 1992; West et al., 1991). Specific assumptions are presented in the supporting information.

The effectiveness of vitamin A supplementation in preventing long‐term hearing loss associated with episodes of purulent ear discharge during the preschool years used estimates taken from a 16‐year follow‐up study of children who participated in the original vitamin A trial (West et al., 1991) conducted by Schmitz et al. (2012), which found a 42% reduction in children with prospectively documented and validated (Katz et al., 1998) ear discharge in their preschool years in Nepal, with other assumptions provided in the supporting information.

Looking ahead, we estimated the potential survival benefit of introducing a neonatal vitamin A prophylaxis programme in Nepal, assuming an average all‐cause mortality reduction of 13% for South Asian settings, as derived from a recent individual participant analysis of trials (Neonatal Vitamin A Supplementation Evidence Group, 2018) and adapted with assumptions about vitamin A supplementation coverage and UNICEF estimates of population size (supporting information).

## 3 RESULTS AND DISCUSSION

In this section, we describe the origins of the National Vitamin A Program in Nepal, its evolution over time (Table 1), and the elements that were critical to its sustained success. We then describe the present‐day situation 25 years later including the opportunities and challenges going forward.

**Table 1:** Timeline of programme implementation

1981	Nepal Blindness Survey shows that the prevalence of Bitot's spots and night blindness, forms of xeropthalmia, exceed WHO cut‐offs for a public health problem
1988	Female community health volunteer programme begins
1990	Nepal commits to improve nutritional conditions for children by 2000 as part of the World Summit for Children
1991	Trial in Sarlahi (1989–1990) shows that biannual VAS reduces mortality among children aged 6–59 months by 30%
1992	Trial in Jumla (1989) shows that biannual VAS reduces mortality among children aged 6–59 months by 26%
	Eighth National Plan (1992–1997) includes VAS as a child mortality prevention strategy and 10‐year National Programme of Action sets a target of achieving virtual elimination of vitamin A deficiency
1993	Implementation of the Nepal vitamin A programme begins in eight districts with financial and technical assistance from USAID and UNICEF, with a plan to scale up in phases.
	The NTAG established to provide technical support to the national vitamin A programme
1993‐2010	Minisurveys conducted following each campaign are used to inform scale up of the programme
1996‐2005	Ongoing conflict within the country threatens the programme but VAS coverage continues to remain high.
1997	Average VAS coverage in programme districts reaches 80%; National Immunization day for polio integrated with vitamin A programme
	Following successful implementation in 32 priority districts, decision is made to expand the programme to all 75 districts of the country
1998	National Vitamin A Survey shows that the prevalence of clinical vitamin A deficiency has fallen but remains a problem
2002	National Vitamin A programme reaches all 75 districts of Nepal
2008	Vitamin A strategic review meeting held, and all activities are mainstreamed under the Government of Nepal Fiscal Year Budget
2015	MoHP begins to fully procure vitamin A capsules with government funds, ensuring sustainability of the programme
2016	National micronutrient survey suggests that vitamin A status has markedly improved but that intake of vitamin A rich foods remains low

Abbreviations: MoHP, Ministry of Health and Population; NTAG, National Technical Assistance Group; USAID, United States Agency for International Development; VAS, vitamin A supplementation; WHO, World Health Organization.

The National Vitamin A Program was forged from strong links between the scientific community, government policymakers, civil society, donors, and local communities. Discussions about how to translate the findings of the two vitamin A child mortality trials into a programme benefitted from the experiences that the research teams had in trying to optimize coverage under two very different settings: mountainous Jumla and the population‐dense, low‐lying plains (*Terai*) of Sarlahi and informed the development of implementation guidelines and a strategy.

### 3.1 Development of the Guidelines for Implementation of the National Vitamin A Deficiency Control Program

First produced by the Nutrition Section in the Child Health Division of the MOH in 1992 and subsequently revised in 1996, the Guidelines for Implementation of the National Vitamin A Deficiency Control Program in Nepal (MOH Nepal, 1996) outlined the objectives, protocols for implementation, a monitoring and evaluation plan, and the roles that different organizations were assigned in combatting deficiency. The guidelines identified 32 priority districts, largely located in the western region of the country and the *Terai*, as being at particularly high risk of xerophthalmia and to be prioritized during the roll‐out of the programme.

The strategy encompassed multiple facets of a national strategy to address vitamin A deficiency (MOH Nepal, 1996) including

1. Preventive vitamin A supplementation twice per year, once in March/April (*Baisakh*) just prior to the time when measles and xeropthalmia were known to peak; and again in October/November (*Kartik)* in the periharvest season, prior to a minor seasonal peak in xerophthalmia (Sinha & Bang, 1973). A dosage of 100,000 IU was recommended for children 6–11 months of age and 200,000 IU for children 12–59 months of age.
2. Nutrition education and efforts to increase the production and consumption of vitamin A‐rich foods.
3. Training of health care workers to treat cases of xeropthalmia, measles, severe malnutrition, and prolonged diarrhoea with vitamin A and supporting efforts to ensure adequate supply of vitamin A capsules at health posts and clinics.

### 3.2 Providing technical guidance as the programme scaled up

Acknowledging the potential difficulty in bringing such a programme to scale, the decision was made to gradually expand the programme by two to eight districts at 6‐month intervals over 9 years. This enabled the local adaptation of the programme under varying contexts and as new challenges were encountered and permitted the scale‐up teams to focus technical assistance on a limited number of districts at a time. To support the technical needs and the learning process, United States Agency for International Development (USAID) funded the creation of the Technical Assistance Group under John Snow International, which was tasked with supporting implementation, learning, and monitoring and evaluation. Later, an NGO, the NTAG, was founded in 1995 by the Nepali Expert Team and supported by UNICEF and USAID to specifically guide, adapt, and monitor implementation of the vitamin A programme on behalf of the MOH.

The National Vitamin A Program in Nepal may have been one of the first nutrition programmes to harness contributions from so many sectors. At the central level, the Ministries of Planning, Agriculture, Education, Local Development, and Women and Social Welfare were among those involved in the planning of the programme, and workers from each of these sectors also supported programme delivery at the community level.

### 3.3 The important role of FCHVs

On the basis of the experiences of the Jumla trial, which had delivered vitamin A supplements to children using a cadre of trained local community workers (both male and female) that had previously been involved in case management of acute respiratory infections (Pandey, Daulaire, Starbuck, Houston, & McPherson, 1991), the decision was made to also rely on a cadre of female community workers to deliver the National Vitamin A Program. The roll‐out of the vitamin A programme required the recruitment of a large cadre of FCHVs, establishment of their responsibilities and training needs, and in particular, cultivating a recognition of their important role within their communities. Although the village health workers were formally charged with ensuring the supply of vitamin A capsules and supervising the administration of the dosing, the FCHVs took the lead in reminding communities and family members about the distribution date, time, and place. They worked very closely with ward members and Village Development Committee chairmen to organize promotional campaigns. The FCHVs also had responsibility of administering the vitamin A supplements to children. Initially, FCHVs received no money, but in 2002, the decision was made to give them a small stipend of NPR 200 per day (approximately $2.5). Over time, NTAG established an FCHV Endowment Fund, with money coming from local Village Development Committees (subdistrict unit) and urban municipalities. In 2014, this was raised to NPR 400 per day (approximately $3.9) to cover their transport‐related costs.

During the weeks leading up to each 6‐monthly campaign, FCHVs toured their catchment areas to update the registers of eligible children, raise awareness of the campaign, and advocate for participation. Activities to mobilize communities varied by types of community. In towns and cities, such as Butwal and Nepalganj, “vitamin A rallies” were organized, involving officials from government agencies including the District Health Authority, Local Development Office, and Police Office, among others. In smaller villages, local women's groups organized rallies with support from local NGOs, community‐based organizations, and health workers. Members from all organizations were enlisted to spread the word. In mountainous villages, these organizations and individuals would use loud speakers to communicate to groups of people the date and venue of vitamin A distribution. In parts of the country, “vitamin A magicians” were even hired to perform magic tricks and stir interest in the programme. During a typical 3‐day vitamin A supplementation programme, vitamin distribution sites were set up at different places during the first 2 days to facilitate attendance, with FCHVs noting compliant children on registers. On the third day, FCHVs sought out registered, nonrecipient children with house‐to‐house visits to maximize coverage.

The vitamin A distribution programme scaled up and sustainably achieved >80% target group coverage across Nepal throughout a decade‐long civil war (1996–2006) that claimed ~17,000 lives and disrupted infrastructure, health services, and virtually all aspects of economic life in the country, a testimony to the programme's advocacy and the dedication, pride, and perseverance of all involved (Devkota, 2005). Although the conflict disrupted logistics, demand from communities was so strong that a “day of tranquility” was negotiated every 6 months to facilitate the national vitamin A campaign (Houston, Mathema, Adikari, & Pokharel, 2006). Knowing the popularity of the programme in communities, Maoist leaders even attended the vitamin A training in many remote areas.

### 3.4 Government and donor commitment and overcoming bottlenecks

In the early days of the programme, a consortium of bilateral and multilateral agencies supported the National Vitamin A Program by providing funding and technical support, including USAID, Canadian International Development Agency, UNICEF, Australian Agency for International Development, and the Micronutrient Initiative. With the strategic review of the vitamin A programme in 2008, the Government of Nepal provided increased financial support for the programme, including registers, scissors to cut the capsules, pens to record the registers, and bottles for repacking the vitamin A supplements locally. The MoHP also took over supervision and monitoring of the programme. As time went on, the government financial commitment grew, and in 2015, the Government of Nepal took full responsibility to run the campaign with an annual allocation of $1,293,510 including the procurement of 10 million vitamin A capsules.

The nationwide delivery of vitamin A capsules and other supplies to distribution sites was channeled through existing Village Development Committee health centres and local infrastructure, with NTAG, other NGOs, and UNICEF monitoring inventories to anticipate and overcome supply bottlenecks. With a system in place that could sustain high coverage across one of the most challenging national landscapes in the world, other health and nutrition services were added to the vitamin A programme “scaffold,” including polio vaccination and anthelmintic treatment. More recently, following the earthquake of 2015, the distribution of multiple‐micronutrient powders (MNP) and screening for acute malnutrition were also integrated into the concept of “Child Nutrition Week,” building on the existing vitamin A programme, and were subsequently expanded as a biannual programme.

### 3.5 The role of monitoring and learning in the success of the programme

The strengths of Nepal's vitamin A supplementation programme were its reliance on monitoring data to anticipate challenges, the development and testing of new approaches to overcome difficulties, and the expansion of solutions to sequential waves of start‐up districts. The technical assistance on monitoring and learning was provided by NTAG and was strongly encouraged by government and supported by USAID and UNICEF to assure sustained high coverage.

A programme monitoring system was put in place with feedback mechanisms through which lists of children that had been dosed were compared against the FCHV registers by health workers following each round of vitamin A distribution. Performance summaries were regularly sent by districts to the MoHP and NTAG, reviewed, and feedback provided to districts during quarterly review meetings. Ensuring this feedback loop was perceived as an essential way of communicating to the field the importance of the programme. In addition, the country conducted “mini surveys,” beginning with the first round of vitamin A supplementation in 1993 and continuing for nearly 13 years to estimate the coverage of the vitamin A supplementation campaigns and accompanying interventions at each round (NTAG, 1999). Notable features of these minisurveys included

- Representative sampling approaches covering all newly introduced districts, including at least one district from each phase of programme expansion.
- Collection of survey data within 1 month after each round of distribution to minimize recall bias and quickly turnaround data to improve the next round of supplementation.
- Collection of process information from FCHVs, which served to identify logistical and other challenges and to provide a feedback loop enabling their voices to be heard.
- Collection of data on the reasons why children were not supplemented and analysis of the factors associated with being missed.
- Collection of data on night blindness.

In the early days of the supplementation programme, these surveys provided rapid data that could be used to assess programme performance. As the programme scaled up, more information was sought from the minisurveys to assess the effectiveness of multiple aspects of community mobilization including use of radio and TV, loud speakers, and leaflets in terms of the proportion of people at the community level that reported hearing about the campaign from each of these different sources.

Over time, the country also showed its willingness to pilot new approaches and adjust policies to improve coverage and protect children from vitamin A deficiency. When Demographic and Health Survey findings showed that infants aged 6–11 months tended to have much lower coverage than older children (MOH, 2002; MoHP, 2007; Thapa, 2010), the decision was made to provide FCHVs with instructions to supplement infants with 100,000 IU as soon as they turned 6 months of age rather than wait for the next round of the 6‐monthly campaigns. If children were missed at 6 months of age, they were dosed at 9 months by FCHVs during the routine measles vaccination. This policy was possible because the FCHVs were based in the communities themselves. In the three areas where this approach was piloted, coverage among children aged 6–8 months increased from 56% in 2012 to 70% in 2013 (Public Health and Infectious Disease Research Center, 2014).

Early programme reviews also raised the concern that children missed by the programme tended to come from poorer households and emphasized the need to improve the equity of the programme (Fiedler, 2000). Such findings were of particular concern given epidemiologic evidence that xeropthalmia tends to cluster in poorer households (Khatry et al., 1995). Multivariable analysis of 2006 Demographic and Health Survey data found that children from the poorest quintile were 35% less likely to receive a vitamin A supplement in the previous 6 months compared with those from the richest quintile after adjusting for factors such as education, urban/rural residence, gender, and child age (Thapa, 2010). Although a similar multivariable analysis has not been conducted over the past 9 years, unadjusted analyses of the Demographic and Health Survey from 2006 suggest similar coverage for the wealthiest and poorest quintiles has been maintained over time (Table 2).

**Table 2:** Percentage of children aged 6–59 months who received a vitamin A capsule in the past 6 months by wealth quintile and location

	Vitamin A capsule coverage by year, %		
	2016	2011	2006
Wealth quintile			
1st	89.9	89.4	84.9
2nd	85.6	89.7	87.7
3rd	83.2	91.4	90.5
4th	85.7	91.3	90.0
5th	87.4	90.8	84.8
Location			
Urban	85.4	86.4	80.6
Rural	87.4	90.8	88.5

*Note.* Data from the 2006, 2011, and 2016 Demographic and Health Surveys.

The urban programme began much later in Dharan in 1995. The community awareness observed in rural areas was much more difficult to achieve in urban areas because there were no FCHVs at the start of the urban programme. Instead the vitamin A programme relied on volunteers recruited for the National Polio Programme, who had not received the same training as the FCHV's. Additional challenges faced by the programme in urban areas included (a) perceptions by urban residents that they did not need to receive vitamin A due to their better diet and (b) less intensive promotion campaigns. Despite these challenges, the rural–urban coverage gap has also narrowed over time (Table 2).

### 3.6 What has the impact of the programme been?

A number of prior efforts have quantified the effectiveness of the programme in terms of the lives saved and the cost effectiveness of the programme. An analysis of data from the 2001 Demographic and Health Survey estimated that the mortality reduction among children aged 6–59 months was 53% in programmes, a number that surprisingly exceeded the ~30% efficacy observed in trials (Thapa et al., 2005). The author of the study attributed such findings to the inclusion of other inventions and services provided by FCHVs alongside the vitamin A supplements. A separate analysis conducted using data from the start‐up of the programme in Nepal found that the programme cost ranged from $327 to $397 per death averted under different coverage assumptions, placing it among the most cost‐effective health interventions (Fiedler, 2000). An even lower estimate of $11 per death averted was calculated on the basis of experiences in the Jumla trial (Daulaire et al., 1992).

**Figure 1:**
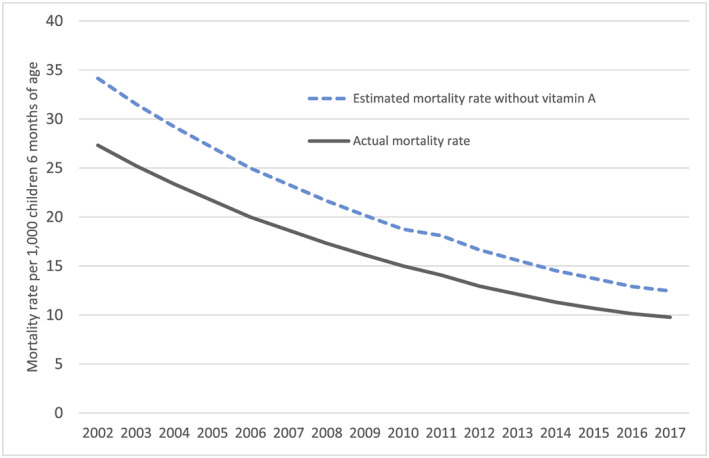
Comparison of actual mortality rates versus estimated mortality rates in absence of preventive vitamin A supplementation among children aged 6–59 months, 2002 to 2017

†

**Figure 2:**
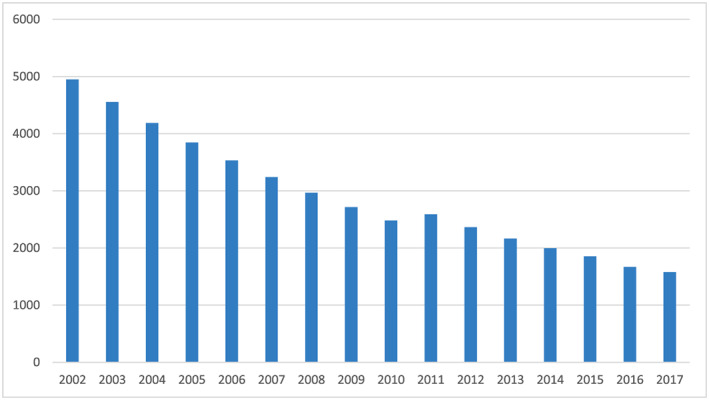
Estimated number of lives saved each year due to vitamin A supplementation in Nepal, 2002 to 2017

As illustrated in Figure 1, assuming that the underlying risk environment (dietary intake and infectious diseases) has remained stable over time, the mortality rate among children aged 6–59 months in Nepal would have been much higher in absence of an effective vitamin A programme. Coverage rates as measured by surveys have ranged from 81–92%, and we estimate that a total of 46,675 child lives were saved between 2002 (when the programme went national) and 2017 (Figure 2). Even in the lower mortality environment that Nepal now has compared with the start of the programme, 1,578 lives per year, or ~22% of 6–59‐month old deaths, may be saved through preventive vitamin A supplementation, with additional lives saved through the treatment of xeropthalmia and measles.

Additional benefits of the programme may also be present in terms of preventing disabilities such as blindness and hearing loss. A recent finding on the basis of the follow‐up of children who participated in the vitamin A supplementation trial in Sarlahi as preschoolers was a 42% reduction in the risk of hearing loss attributable to otitis media in adolescence and young adults (Schmitz et al., 2012). Applying these findings to modern day population estimates suggests that each year the vitamin A supplementation programme may be saving 4,593 cases of hearing loss from middle ear infections (supporting information).

### 3.7 Changes in vitamin A status over time

The 1998 National Micronutrient Status Survey suggested that nearly 17% of children aged 6–59 months had subclinical vitamin A deficiency, as measured using serum retinol (Table 3; MOH, 1998). By 2016, the prevalence of children with deficiency was assessed at 8.5%, as measured using retinol binding protein and 4.2% according to the modified relative dose response assay (Ministry of Health and Population, New Era, UNICEF, EU, CDC, 2018). The apparent drop in deficiency in this age group might be expected if serum retinol had improved due to supplementation. However, low levels of deficiency (<3% had a modified relative dose response assay > 0.060) observed among women in the 2016 survey are difficult to explain given that this group did not receive supplementation. Evidence from other settings also suggests that serum retinol rises in response to supplementation only transiently (within 2 months following receipt) but does reflect underlying dietary consumption of vitamin A‐rich foods (Palmer, West, Dalmiya, & Schultink, 2012). As such, the findings of the most recent survey are somewhat paradoxical, given the evidence outlined below that vitamin A‐rich foods are not consumed frequently in Nepal. Additional research may be needed to contextualize these findings in relation to the dietary intake of vitamin A in Nepal.

**Table 3:** Findings of the most recent two micronutrient status surveys in Nepal^a^

	1998 National Micronutrient Status Survey (95% C.I)	2016 National Micronutrient Status survey (95% C.I.)
**Child (6‐59 months)**		
Serum retinol<0.7micromol/L	16.6%	12.5% (9.8, 16.0)
MRDR^b^, geometric mean (SD)	0.013 (2.798)	
Vitamin A deficiency MRDR >0.060		4.2% (2.4, 7.1)
Retinol binding protein <0.69 micromol/L		8.5% (6.7, 10.6)
Night blindness	0.27%	
Bitot's spots	0.33%	
**Women (15‐49 years)**		
MRDR, geometric mean (SD)		0.010 (3.876)
MRDR >0.060		3.0% (1.6, 5.5)
Current night blindness	4.7%	
Night blindness during last pregnancy	16.7%	8.5% (6.7, 10.6)
Night blindness during last pregnancy without day vision problems		3.2% (2.0, 4.8)

^a^ Sources: (Gorstein, Shreshtra, Pandey, Adhikari, & Pradhan, 2003; Ministry of Health and Population, Nepal, New Era, UNICEF, USAID, CDC, 2018).

^b^ Modified relative dose response.

### 3.8 Progress made on fortification and dietary diversification

Many country‐level and global plans to combat vitamin A deficiency describe three complementary strategies: vitamin A supplementation, fortification, and dietary diversification. From its inception, the National Vitamin A Deficiency Control Program of Nepal outlined two of these three strategies: vitamin A supplementation and the need for behaviour change to “increase dietary vitamin A intake of the target group through nutrition education, increased home production, consumption and preservation of vitamin A rich foods, proper breastfeeding and child feeding practices” (MOH Nepal, 1996).

Compared with vitamin A supplementation, little attention has focused on assessing progress related to dietary diversification or fortification in‐country. However, the minisurveys conducted over time demonstrated a remarkable increase in the proportion of mothers able to identify sources of food rich in vitamin A or provitamin A carotenoids. According to the minisurvey conducted in April 1997, just 51% of mothers could correctly state at least one dietary source of vitamin A (NTAG, 1999). Subsequent minisurveys documented tremendous increase in this metric, often exceeding 90% (NTAG, 1999).

The extent to which this knowledge translated into improved consumption by the target groups remains largely uncertain. Although a number of dietary surveys have been done over time in Nepal, few have used comparable methods, so it has been difficult to assess how intake of vitamin A has changed in the country. A survey from 1998 using the 24‐hr vitamin A semiquantitative method found that the median intake of vitamin A among preschool children was 175 RE (Retinol Equivalents) per day with just 41.7% of the children meeting their minimum requirements for the previous day and the 84.8% of the intake coming from vegetable sources (MOH, 1998). More recent studies have examined the frequency of consumption of different sources of vitamin A in Nepal among preschool aged children and women and suggest that consumption of vitamin A sources of food remains low (Abarca‐Gómez et al., 2017; Dorsey et al., 2018). The proportion of children 6–23 months of age meeting minimum dietary diversity in recent surveys was reported as 45.8% in the 2016 Nepal National Micronutrient Status survey and 47% in the 2016 Demographic and Health Survey (MOH Nepal & ICF, 2017).

Historically, with the exception of iodized salt, fortification efforts in Nepal have been held back for a number of reasons including the difficulty in identifying suitable food vehicles that meet requirements such as centralized processing facilities, market penetration, consumption by target groups, and quality control. The country did enact voluntary fortification of flour processed through roller mills with vitamin A, iron, and folic acid in 2008 and mandatory fortification in 2011, although enforcement has been challenging given open borders with India, and coverage is only estimated at 35% (MoHP, 2018).

### 3.9 Additional scientific discoveries related to vitamin A made in Nepal

Research in Nepal has contributed to other scientific contributions on vitamin A, including findings that maternal supplementation with vitamin A in pregnancy was associated with lower maternal mortality in Nepal and that maternal night blindness in pregnancy increases the risk of mortality and morbidity and is treatable with vitamin A (Christian, West, Khatry, Katz, LeClerq, Pradhan, & Shrestha, 1998a; Christian, West, Khatry, Katz, Shrestha, Pradhan, et al., 1998b; Christian, West, Khatry, Katz, et al., 2000; Christian, West, Khatry, Kimbrough‐Pradhan, et al., 2000; Katz et al., 1995). These findings have drawn attention to the need to address maternal as well as child vitamin A deficiency as a public health problem.

### 3.10 From controlling to eliminating vitamin A deficiency in the era of the Sustainable Development Goals: The way forward

Twenty‐five years after the initiation of the National Vitamin A Programme in Nepal, the country faces a very different landscape than before, with new opportunities and challenges in the fight against vitamin A and other micronutrient deficiencies. Some elements of the shift underway in Nepal of relevance to micronutrient deficiencies include

- Approval of the Multisectoral Nutrition Plan II (MSNP‐II) 2018–2022 by the Government of Nepal in 2017, outlining targets for increased production of dietary sources of vitamin A (meat, milk, fruit, and vegetables), expanding coverage of MNP, promotion of milled and fortified flour, as well as sustained coverage of vitamin A supplementation to children aged 6–59 months.
- A recent restructuring of government systems under a new federalist structure, with greater decentralization of procurement and programme management including for vitamin A.
- Recent findings from the National Micronutrient Status Survey, 2016, suggesting potential changes in the prevalence of subclinical vitamin A deficiency among children and (MoHP, 2018)
- Rapidly growing mobile phone access and opportunities for new channels of behaviour change communication.
- Expanding roads and market access by many but not all.
- Demographic shifts including a rise in single female‐headed households and men sending remittances from a distance.
- Growing availability and consumption of highly processed foods.
- Newly available fortification technologies including biofortified foods and rice fortification.

Reflecting on potential policies to pursue going forward, it is worth noting that even in high‐income countries, multiple strategies have been needed to alleviate micronutrient deficiencies. As described above, the MSNP to guide action from 2018–2022 contains elements related to each of the three pillars of vitamin A deficiency control (supplementation, fortification, and dietary diversification). In this section, we examine the current status and future opportunities related to these deficiency control strategies, and implications given current realities, with a view towards the next 25 years.

### 3.11 Dietary diversification

The MSNP‐II sets concrete targets for the increased production of vitamin A‐rich foods and outlines the need to scale up behaviour change communication efforts. A number of projects are currently underway with explicit goals of diversifying the diets of infants and young children in Nepal, particularly those in food insecure areas, which may help to inform the question of how to best achieve these targets. The Suahaara II project, funded by USAID, aims to increase the production and consumption of micronutrient‐rich foods, and findings reported to date suggest positive results (Suresh et al., 2019), although it is well known that dietary diversification tends to occur slowly and often requires complementarity with other approaches to vitamin A deficiency control. Increasing access to mobile phones represents an opportunity for behaviour change communication that Suahaara II and other projects are already using, and the effectiveness of this approach should be studied. Going forward, it will be important to draw on lessons from Suahaara II as it is the largest project to date to aim for dietary diversification at scale in Nepal.

### 3.12 Supplementation programmes

The national vitamin A supplementation programme has maintained impressively high coverage over time. This coverage has been sustained in recent years despite less investment in training of FCHVs, promotional activities, minisurveys, or other aspects of the programme. As a result of decentralization, the programme faces both new opportunities and challenges. Given the community‐based delivery platform for vitamin A supplementation, implementation of the programme may not be adversely affected by decentralization, provided supplements are delivered to the field on time. One challenge in the short term is that the responsibility for supply chain management, including the procurement of vitamin A supplements, has been transferred from the central government to the province level. There are likely to be capacity challenges at the provincial level, at least in the short term, to procuring vitamin A supplements. It may make more sense to readjust responsibilities so that the provinces procure vitamin A capsules from the central government or UNICEF using government funds until they are able to independently manage this process. Decentralization may also have certain advances; flexibility of funding may enable provinces with lower coverage (such as Province 2 that had a vitamin A supplementation coverage of only 77.6% in 2016 vs. a national coverage rate of 86.3; MOH Nepal & ICF, 2017) to develop and test approaches to overcome the specific challenges faced in attaining high coverage in the area and resurrecting the minisurvey approach to measure the effectiveness of those approaches. To do so will require that the vitamin A programme receives a high priority at the province level, and awareness building among local policy makers may be needed.

### 3.13 Neonatal vitamin A: A new intervention for tackling the high burden of neonatal mortality?

Nepal made tangible progress in reducing the burden of under‐five mortality during the Millennium Development Goal (MDG) era, surpassing its 2015 goal of 54 deaths per 1,000 live births by attaining U5MR of 38 deaths per 1,000 live births by 2014 (Government of Nepal National Planning Commission, 2015), It is likely that vitamin A supplementation of children aged 6–59 months played an important role in this achievement. The MDG infant mortality target of 36/1,000 live births was also surpassed by 2014 with an achievement of 33/1,000 live births.

Going forward, Nepal has an ambitious target of reducing neonatal mortality to a rate of <10/1,000 live births by 2030 (Government of Nepal National Planning Commission, 2015). Achieving this target will require additional efforts to address the existing causes of neonatal mortality. Epidemiological analyses of the causes of neonatal deaths in two sites in Nepal (Dhanusha and Makwanpur) suggest that infections remain an important cause, responsible for 40% of deaths, with neonatal pneumonia responsible for 25–29% of deaths (Fottrell et al., 2015).

Since the first trial of neonatal vitamin A supplementation in Indonesia was published in 1996, 11 placebo‐controlled trials have been published, five in Asia and six in Africa. Although there has been no benefit to survival reported from the trials in Africa, neonatal vitamin A supplementation was found to significantly reduce 6‐month mortality in the trials conducted in Southern Asia (RR 0.87; 95% CI [0.77, 0.98]; West et al., 2018). Assuming a similar mortality benefit, if the intervention were introduced in Nepal and implemented at 80% coverage, it could reduce mortality among infants less than 6 months by 10%, saving approximately 1,400 lives per year among infants less than 6 months. Prior operational research conducted in four sites in Nepal helps to inform a potential delivery modality for such a programme, as it suggested that delivery through FCHVs rather than through home delivery kits led to higher coverage rates (62% vs. 45%; Klemm, 2011). Neonatal vitamin A supplementation seems like an obvious opportunity for the country to pursue.

### 3.14 Fortification programmes

The 2018 National Micronutrient Survey provided a number of findings of relevance to fortification policy. Fortified complementary foods were consumed by only 13.4% of children aged 6–23 months and 6.6% of children aged 6–59 months, although interestingly nearly 30% of children aged 6–8 months consumed them. Consumption was also greater among higher castes (Brahmin, Newar, and Chetri). Consumption of sweets was 75.1% and sugar‐sweetened beverages was 21.8% among children aged 6–59 months in the past 24 hr; findings suggest the possibility that purchasing power could be redirected towards healthier complementary foods.

Nearly 95% of children aged 6–59 months had consumed cooking oil, suggesting that it may be an optimal vehicle for fortification with vitamin A. Given that the majority of vegetable oil consumed in Nepal was imported according to 2013 data from the Food and Agriculture Organization *Food Balance Sheets* (Food and Agriculture Organization Corporate Statistical Database, 2019), it will be important that fortification policy also addresses imported vegetable oil.

Recent efforts spearheaded by World Food Programme have aimed to introduce fortified rice, initially through the social safety net programmes. This approach seems promising given the high consumption of rice nationally, although perhaps less effective for farming populations that grow their own rice.

Additionally, the MSNP‐II outlines the expansion of MNPs as an important intervention for controlling vitamin and mineral deficiencies in Nepal. Lessons learned from a pilot study implementing MNP distribution as part of a comprehensive IYCF intervention in two districts of Nepal will be important to consider when expanding this programme (MoHP, New Era, UNICEF/Nepal, CDC, 2018). The study found strong uptake of knowledge about the product (termed *Baal Vita*) but found that sustaining adherence over time was challenging. Further, formative research may help to identify ways of improving adherence as the programme expands.

## 4 CONCLUSIONS

The National Vitamin A Program of Nepal is viewed by many in Nepal as the most successful public health intervention, likely contributing substantially to the acceleration of child mortality reductions and the achievement of the mortality MDG. What facilitated the success of the National Vitamin A Program? Clearly there were multiple elements that contributed, but in the end, it boils down to the motivation, trust, and drive of people at all levels of the country, from families and communities to the very top, to achieve the result of getting a capsule in the mouths of every eligible child twice a year. Certainly, it would not have been possible without government commitment and leadership as well as the external backstopping and technical support provided by the international community and donors over two decades. Also key was the fact that programmers were able to create a demand for the capsule that compelled those responsible for managing the vitamin A capsule supplies to ensure that sufficient capsules were delivered to all distribution sites on time. Integral to creating this demand was the placement of FCHVs at the heart of the programme and the successful advocacy and community mobilization that turned vitamin A supplementation days into a platform for the delivery of a package of health and nutrition interventions. Going forward, as the country expands fortification efforts and dietary diversification, it will be important to ensure that sufficient dietary intake of vitamin A exists prior to any scaling down of the programme and that careful monitoring is done during such a transition to ensure that rises in the signs and symptoms of vitamin A deficiency (which may be a sentinel indicator of increased mortality) do not occur.

## CONFLICTS OF INTEREST

The authors declare that they have no conflicts of interest.

## CONTRIBUTIONS

AT‐L and KPW drafted the article and conducted analysis. NP, DLM, and SC contributed to the article through provision of reference material, editorial support, and drafting of some policy sections of the main text. Mr. KRP contributed to the article through editorial support and drafting of some policy section of the main text. Dr. (hon) RKS contributed to the articles through reference material, editorial support, and drafting of the article.

## Supporting information

Data S1 Supporting information

## Footnotes

†
Data sources: UN Interagency Group for Mortality Estimation (IGME, 2018), DHS and micronutrient surveys for vitamin A capsule coverage, calculations presented in supplementary materials
