# Supplementary material for: To see, hear, and live: 25 years of the vitamin A programme in Nepal
Source: Matern Child Nutr. 2020 Feb 27;18(Suppl 1):e12954. doi: 10.1111/mcn.12954 (PMC8770656; doi:10.1111/mcn.12954)
Supplement: Supplementary file 1 — Data S1 Supporting information [file MCN-18-e12954-s001.docx]

**Supplementary materials**

***Data and calculation for Figure 1***

***
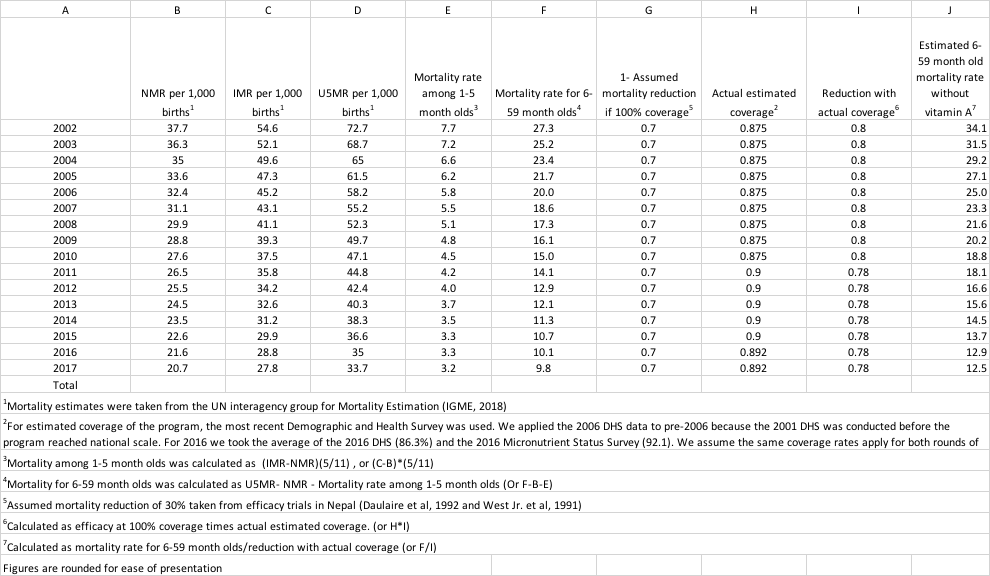
***

***Data and calculation for Figure 2***

**
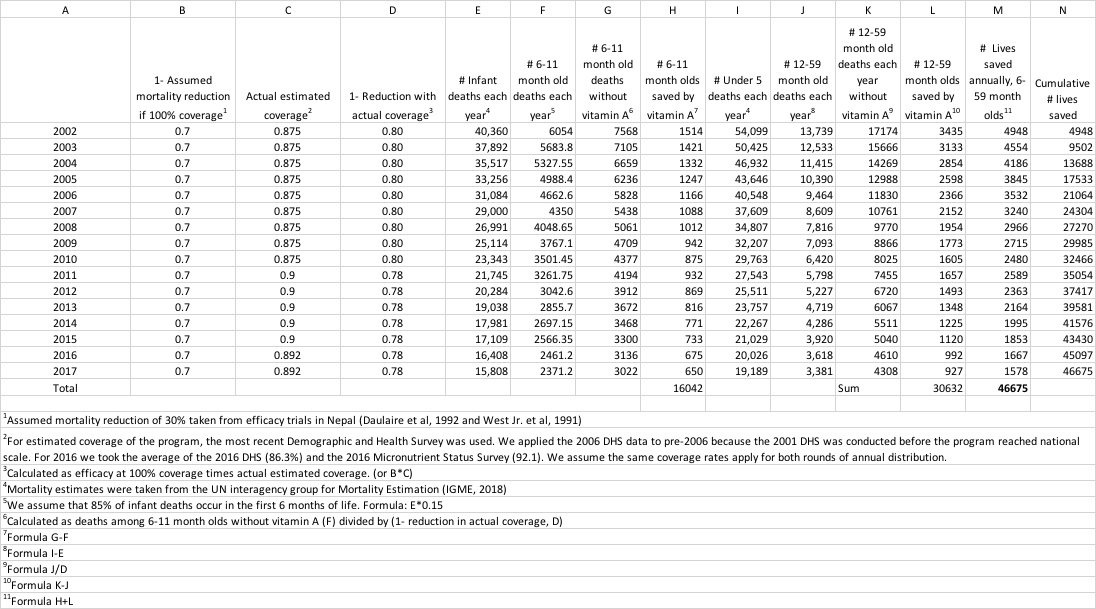
**

**Calculation of number of cases of hearing loss averted**

Population of under 5 children: 1.45 million (UN Statistics)

Number of otitis media cases by 5 y (19.2%): 278,980 (Schmitz et al., 2012)

# with hearing loss if not supplemented by VAC (19.6%): 54,680 (Schmitz et al., 2012)

# With hearing loss with a 42% reduction with VAC: 31,714 (Schmitz et al., 2012)

# Hearing loss cases averted per 5 years: 22,965

Estimated # hearing loss cases from middle ear infections being prevented each year through VAC program: 4,593
